# Study on Foaming Quality and Impact Property of Foamed Polypropylene Composites

**DOI:** 10.3390/polym10121375

**Published:** 2018-12-11

**Authors:** Wei Gong, Hai Fu, Chun Zhang, Daming Ban, Xiaogang Yin, Yue He, Li He, Xianglin Pei

**Affiliations:** 1 College of Materials and Architectural Engineering, Guizhou Normal University, Guiyang 550025, China; gw20030501@163.com (W.G.); fullsea@yeah.net (H.F.); bdaming@gznu.edu.cn (D.B.); m13885115516@163.com (X.Y.); jhb20140101@163.com (Y.H.); 2 National Engineering Research Center for Compounding and Modification of Polymeric Materials, Guiyang 550014, China; zhangchun925@126.com

**Keywords:** polypropylene, foaming quality, impact property, intrinsic toughness

## Abstract

In the present work, foamed polypropylene (PP) composites were prepared by chemical foaming technology, and the foaming quality and impact property of the foamed PP composites were studied. The results showed that the foaming quality was significantly improved after the introduction of thermoplastic rubber (TPR) and polyolefin elastomer (POE). Meanwhile, it was found that the impact property depended on the intrinsic toughness and contribution of foams (cells) to the PP composites. Furthermore, the data regarding impact property in low temperature showed that when the temperature was between −80 and −20 °C, the impact properties of the foamed PP composites were higher than that of the unfoamed sample, which was due to the impact property being completely contributed by cells under this condition. Meanwhile, when the temperature ranged from −20 to 20 °C, the impact property of the unfoamed sample was higher, which was due to the PP matrix contributing more to the impact property under this temperature. This work significantly improved the foaming quality of foamed PP composites and provided reliable evidence for the improvement of impact property.

## 1. Introduction

Nowadays, the microfoamed polymer materials [1,2,3] have attracted increasing attention due to their excellent comprehensive properties. Compared with traditional foamed materials, the microfoamed polymer materials possess highly specific properties, including good thermal stability, excellent sound absorption property, low thermal conductivity and dielectric constant, etc. [4,5,6]. As a result, microfoamed polymer materials have been widely used in transportation, the military industry, aerospace, electronics, daily necessities, and so on [7,8,9,10]. When the microfoamed polymer materials are used as structural materials, the comprehensive properties of the materials need to be higher, especially the mechanical properties, because the foamed materials have a certain weight loss [8,11]. Generally, the mechanical properties of the microfoamed polymer structural materials are mainly determined by the morphology of cells in the material, such as the shape, diameter, density, and distribution of the cells [11,12]. Therefore, further study of the foaming quality and mechanical properties, such as the impact property of the microfoamed polymer materials, is worthwhile. On the other hand, isotactic polypropylene (iPP), as a widely used semicrystalline polymer, has a considerable commercial importance owing to its numerous advantages, such as low cost, easy processing, recyclability, and excellent mechanical performances [13,14,15]. iPP also has various crystalline modifications, such as α, β, γ, etc. [16]. Due to its excellent comprehensive properties, it is usually produced into various forms of polymer products [16].

Recently, more and more researchers have focused on the research of foamed polymer materials, especially the PP-based materials. Rizvi et al. [17] fabricated a microfoamed PP/PTFE material, and the formative fibrous PTFE significantly improved the foaming quality with higher bubble density and volume expansion ratio. The foamed PP/PTFE composites exhibited a high sorption capacity to CO_2_. Keramati et al. [18] also studied the effects of nanoclay on the foaming behavior of PP/ethylene–propylene–diene monomer rubber (EPDM). They found that the added nanoclay was dispersed evenly in the PP matrix, and a small amount of nanoclay could hugely increase the cell density and reduce the cell size. Kuboki et al. [19] have explored the effects of cellulose content and processing condition on the foaming quality and mechanical properties of the PP foamed composites reinforced with fiber by injection molding, and the results showed that the strength, flexural modulus, and notched impact property increase with the increase of cellulose content. Xi et al. [20] also fabricated a microcellular foamed PP/GF composites, and found that the introduction of GF (glass fiber) could improve the foam structure and mechanical properties of the foamed materials. The above reports aim at the preparation of foamed materials by introducing materials such as nanoparticles, fibers, EPDM, polyolefin elastomer (POE), etc. into a polyolefin material, further studying the effects on the foaming quality and mechanical properties of the foamed materials. These methods of preparing foamed materials mainly focus on the physical foaming, while chemical foaming technology is rarely reported.

In this work, the foamed PP composites are prepared by adding different types of elastomers using the chemical foaming technology. The effects of foaming quality and the impact property of the materials are systematically studied through their structures, crystallization behavior, rheological behavior, low-temperature toughness, and so on.

## 2. Materials and Methods

### 2.1. Materials

PP (iPP), S1003 was purchased from China Sinopec (Shanghai, China) with a melt flow index of 3 g·10 min^−1^ at 230 °C and a density of 0.92 g·cm^−3^. PP-MAH and CA100 was obtained from Arkema, Paris, France with a grafting rate of 1% and melt flow index of 10 g·10 min^−1^ at 230 °C. TPR elastomer 2095 was gained from Shenzhen Jiaxinhao Plastic Products Co., Ltd., Shenzhen, China. POE elastomer 8200B with a melt flow index of 5 g·10 min^−1^ at 190 °C and density of 0.87 g·cm^−3^ was obtained from Dupont Corporation, Wilmington, DE, USA.

### 2.2. Preparation of Modified Materials

First of all, the PP and the elastomer were melt extruded at a ratio of 9:1 on a twin-screw extruder (CTE20, Coperion Koryo Nanjing Machinery Co., Ltd., Nanjing, China) to obtain the masterbatch, and the parameters of the extrusion process were as follows: temperature of 180–200 °C, screw speed of 100 r·min^−1^, feeding speed of 10 r·min^−1^. Then, the PP composites were prepared by melt blending the above masterbatch and PP on a twin-screw extruder with an extrusion temperature of 180–200 °C and a screw speed of 200 r·min^−1^.

### 2.3. Preparation of Foamed Sample

**Figure 1:**
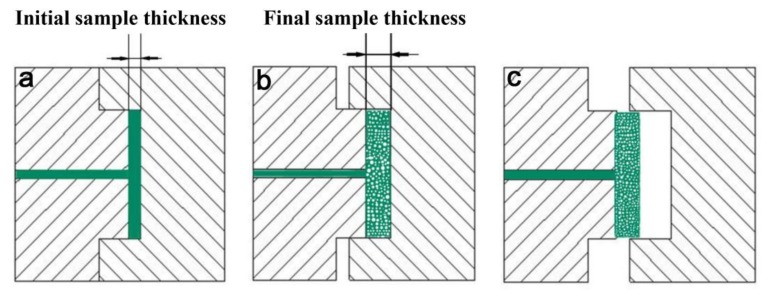
The schematic of the foaming process with adjustable volume: (**a**) injection process, (**b**) slightly open the mold to depressurize, (**c**) open the mold.

PP foamed samples were prepared by the microcellular injection-foaming molding machine equipped with a volume-adjustable cavity (Figure 1). The extrusion temperature profile from the hopper to nozzle was 165–175 °C, and the expansion ratio was kept at a constant, and controlled by the thickness of the sample expanding from 3.5 to 4.0 mm. In this study, the masterbatch and activator were used at 10 and 5 wt % levels, respectively [11,21].

### 2.4. Characterization

#### 2.4.1. Dispersion Characterization of Elastomers

Dispersion characterization of the elastomers was observed with two scanning electron microscopy (SEM) at an accelerating voltage of 10.0 kV (Zeiss, Jena, Germany) and 25.0 kV (KYKY-2800B, Shanghai, China), respectively. The mixture of 40 mL of H_2_SO_4_, 13 mL of H_3_PO_4_, 12.5 mL of H_2_O, and two grams of CrO_3_ were used to etch the sample at 97 °C for five minutes. Subsequently, the sample surface was cleaned with a KQ3200E ultrasonic cleaner (Kunshan Ultrasonic Instrument Co., Ltd., Kunshan, China) at 80 °C for 20 minutes, and dried under vacuum at 80 °C for four hours. The samples were freeze-fractured in liquid nitrogen and coated with gold before observation.

#### 2.4.2. Characterization of Cell Structure

SEM was used to observe the morphology of the foamed (cells) samples. Here, the samples were also freeze-fractured in liquid nitrogen and coated with gold before observation. The average size and size distribution of cells in the foamed samples were analyzed with Image-Pro Plus software (Media Cybernetic, Rockville, MD, USA), and the size distribution of cells could be denoted with a distribution coefficient (*S_d_*) and calculated according to the equation of standard deviation, as follows [21,22]:

(1) $$N_{0}={\left[\frac{nM^{2}}{A}\right]}^{\frac{3}{2}}\left[\frac{1}{1-V_{f}}\right]$$

(2) $$V_{f}=1-\frac{\rho_{f}}{\rho }$$

(3) $$\bar{D}=\frac{1}{n}\sum_{i}^{n}D_{i}$$

(4) $$S_{d}=\sqrt{\frac{1}{n}\sum_{i}^{n}{\left(D_{i}-\bar{D}\right)}^{2}}$$

where *n* was the number of cells, *D_i_* was the diameter of a single cell, *D* was the average diameter of cells (μm), $V_{f}$ was the foaming ratio (%), *M* was the magnification factor, *A* was the area of the acquired image (cm^2^), $\rho_{f}$ was the foamed material density (g·cm^−3^), ρ was the unfoamed material density (g·cm^−3^), and $S_{d}$ was the distribution coefficient of cells (μm).

#### 2.4.3. Thermal Analysis

Differential scanning calorimetry (DSC) experiments were performed with a 200F3 instrument (Netzsch, München, Germany) under nitrogen atmosphere. All of the samples were quickly heated up to 220 °C and held for five minutes, followed by cooling down to 20 °C at a rate of 10 °C·min^−1^. The degree of crystallinity was calculated by ($\Delta H_{m}$/$\Delta H_{m}{}_{0}$) × 100%, where $\Delta H_{m}$ was the fusion heat generated by cold crystallization, and $\Delta H_{m}{}_{0}$ was the theoretical fusion heat of 100% crystalline polypropylene (PP) with a value of 207.1 J·g^−1^ [11,21].

#### 2.4.4. Rheological Properties

Dynamic rheological measurements were carried out on a strain-controlled ARES rheometer (TA Co., Wilmington, DE, USA) with a 25-mm parallel-plate geometry and a one-mm sample gap at frequencies from 0.1 to 400 rad·s^−1^ in the linear viscoelastic range (strain = 2%). A rheological temperature scanning test was performed from 220 to 130 °C with a cooling rate of 10 °C·min^−1^, a frequency sweep of five Hz, and a strain of 0.5%. All of the measurements were performed under nitrogen atmosphere to prevent polymer degradation and moisture absorption.

#### 2.4.5. Mechanical Properties

Tensile tests were performed according to ASTM D638-10 at a draw speed of 50 mm·min^−1^. Three-point flexural tests were executed according to ASTM D790-10 at a crosshead speed of 2 mm·min^−1^. Notched Izod impact property tests were carried out using an mechanical testing & simulation (MTS) impact tester, according to ASTM D256-10. A side-edge notch with angle of 458, depth of 2 mm, and tip radius of 0.25 mm was machined on each specimen. Five measurements were taken for each sample to obtain data repeatability.

#### 2.4.6. Dynamic Mechanical Analysis (DMA)

DMA was performed with a Q800 analyzer (TA Co., Wilmington, DE, USA). The double-cantilever mode was selected, and the measurement was carried out on a rectangular cross-sectional bar of 35 × 10 × 4 mm^3^ (length × width × thickness) from −120 to 120 °C at a heating rate of 5 °C·min^−1^, with an oscillatory frequency of one Hz. 

## 3. Results and Discussion

### 3.1. Dispersion of Elastomers in PP Materials

**Figure 2:**
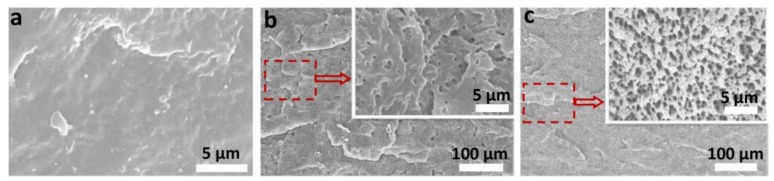
Microstructure of the polypropylene (PP) composites after etching, inset with the partial enlargement: (**a**) pure PP, (**b**) PP/thermoplastic rubber (TPR), (**c**) PP/polyolefin elastomer (POE).

In previous work, in order to make the added elastomers (thermoplastic rubber (TPR) or POE) disperse evenly, we explored the optimum addition ratio of elastomers with the value of about 20 wt % [23]. As shown in Figure 2, neither TPR nor POE particles agglomerate in the PP matrix and exhibit good dispersion. The PP/TPR presents a “sea-island” structure, and the TPR particles show a smaller size distribution (Figure 2b). Meanwhile, for the PP/POE composites, the POE and PP matrix exhibit two continuous interlocking structures (Figure 2c). The well-dispersed second phase (TPR or POE) can supply large numbers of interfacial heterogeneous nucleation sites in the PP matrix, which can significantly increase the nucleation sites of cells and facilitate cell generation [22,24]. Moreover, the good dispersion of the elastomer may have a significant role in promoting the viscoelasticity and impact property of the PP composites.

### 3.2. Influence of Elastomers on Foaming Quality of Foamed PP Composites

**Figure 3:**
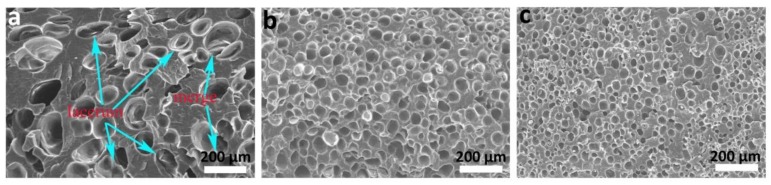
Microstructures of the foamed PP composites: (**a**) pure PP, (**b**) PP/TPR, (**c**) PP/POE.

Further, the foaming quality for the PP composites was measured. It can be seen from Figure 3 and Table 1 that the foaming quality is obviously improved after adding TPR or POE (20 wt %). The number of cells are significantly increased with the values of 7.54 × 10^6^ cells·cm^−3^ and 12.5 × 10^6^ cells·cm^−3^, and the average diameter of cells decreased to 31.21 μm (TPR) and 25.42 μm (POE), respectively. In contrast, for the foamed pure PP, the diameter of the cells is large, and the pore size distribution is uneven and accompanied with bubble merging and rupture (as indicated by the arrow in Figure 3a). Therefore, the addition of TPR or POE into PP materials could significantly improve the foaming quality.

### 3.3. Effect of Elastomers on Crystallization and Rheological Behaviors of the PP Composites

**Figure 4:**
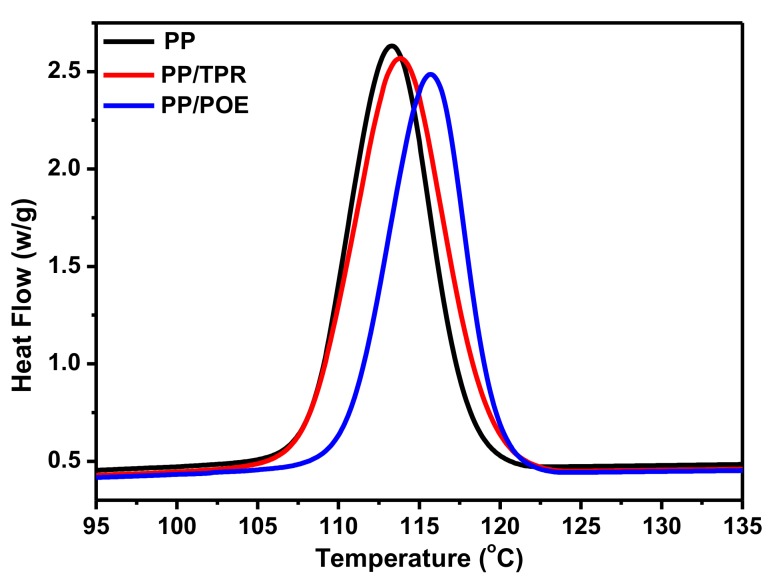
Crystallization curves of the PP composites.

The good foaming quality prompted us to study the crystallization and rheological behaviors of the PP composites. DSC analysis of Figure 4 and Table 2 showed that the initial/peak crystallization temperatures for all of the PP composites increased due to the addition of TPR or POE (20 wt %). As the rapid growth and stabilization process of cells are closely related to temperature, the increase in the initial crystallization temperature can inhibit the deformation and merging of the cells in the later growth period, thus perfecting the cell structures. Meanwhile, the addition of TPR or POE also leads to a decrease in the crystallinity of the PP composites (Table 2), which effectively improves the gas diffusion and increases the uniformity of the cells. Both of these factors led to a significant improvement in the foaming quality.

**Figure 5:**
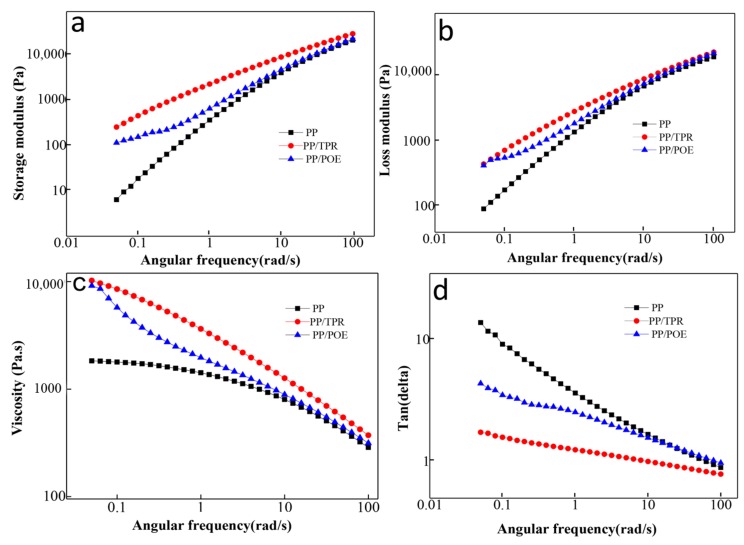
Rheological behavior curves of the PP composites: (**a**) storage modulus, (**b**) loss modulus, (**c**) loss factor, and (**d**) loss factor.

Figure 5 shows the curves of storage modulus (G’), loss modulus (G”), and loss factor for PP, PP/TPR-20 wt %, and PP/POE-20 wt %. Figure 5a shows that the addition of TPR or POE increases the G’ of the PP matrix, and the PP/TPR composites possess the largest G’ value. Usually, in the low-frequency region, only the long relaxation time contributes to the elastic behavior; hence, the G’ of PP/TPR and PP/POE is higher than that of PP. Figure 5b shows that the G” of PP/TPR and PP/POE in the low-frequency region is higher than that of PP. The G” is a key index for material viscosity. Generally, the greater the loss modulus of the material, the better the viscosity of the material [24,25,26,27]. Moreover, Figure 5c further indicates that the viscosity of PP/TPR and PP/POE is higher than that of PP in the low-frequency region. Figure 5d shows the loss tangent versus angular frequency graph. The loss tangent is defined as tanδ (= G”/G’), which reflects the relative ratio of material viscosity to elasticity. It can be seen from Figure 5d that the values of tanδ for the PP/TPR and PP/POE composites in the low-frequency region are smaller than that of pure PP, which indicates that the addition of an elastomer improves the elastic property of the pure PP. These results show that the presence of elastomers increases the viscoelasticity of PP/TPR and PP/POE, and the response of viscoelasticity helps prevent the merger, rupture, and growth of cells during cell forming [22,28]. Therefore, the foamed PP/TPR and PP/POE composites have better foaming qualities.

### 3.4. Influence of Elastomers on the Impact Properties of PP Composites

**Figure 6:**
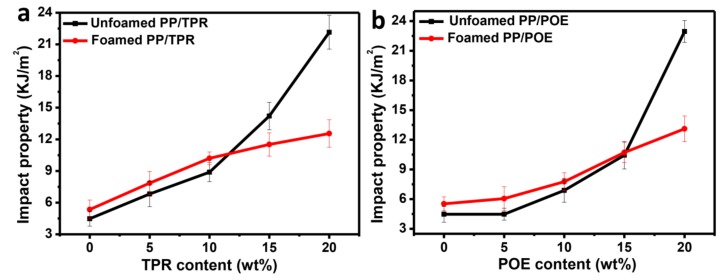
Influence of elastomers on the impact properties of PP composites: (**a**) PP/TPR, (**b**) PP/POE.

**Figure 7:**
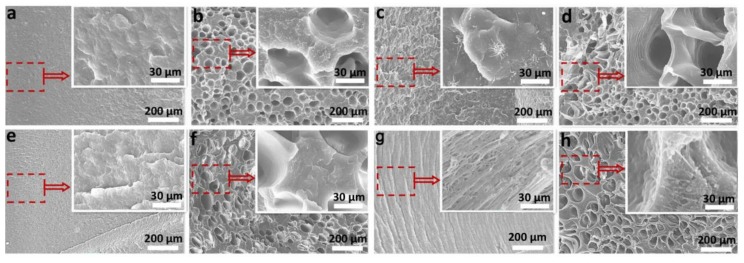
The impact section structures of PP composites, inset with the partial enlargement: (**a**) unfoamed PP/TPR-5 wt %, (**b**) foamed PP/TPR-5 wt %, (**c**) unfoamed PP/TPR-20 wt %, (**d**) foamed PP/TPR-20 wt %, (**e**) unfoamed PP/POE-5 wt %, (**f**) foamed PP/POE-5 wt %, (**g**) unfoamed PP/POE-20 wt %, (**h**) foamed PP/POE-20 wt %.

The good foaming quality also encouraged us to study the impact properties of PP composites. Figure 6 shows the effect of different elastomer contents on the impact property of the foamed and unfoamed PP composites. For the PP/TPR composites, the impact property of both foamed and unfoamed samples gradually increases with the increasing content of TPR (Figure 6a), and the fracture characteristics gradually become rough (Figure 7a–d, here PP/TPR-5 wt % and PP/TPR-20 wt % are used as examples), indicating the gradual increase in material toughness. When the content of TPR is more than 11 wt %, the impact property of the unfoamed sample is higher than that of foamed sample, while when the content of TPR is below 11 wt %, the impact property of the foamed sample is higher. For the PP/POE composites, as shown in Figure 6b, the notched impact property of the foamed and unfoamed samples tended to increase with the increase of POE content, and the fracture characteristics (figures 7e–h, similarly, PP/POE-5 wt % and PP/POE-20 wt % are used as examples) also gradually became rough. When the POE content was higher than 15 wt %, the impact property of the unfoamed sample was greater than that of the foamed sample, but when the POE content was lower than 15 wt %, the impact property of the unfoamed sample was higher. Interestingly, the relationship between the impact property and TPR/POE content has the same relationship as that with the material impact property, of about 10.44 KJ·m^−2^. When the impact property of the material is less than about 10.44 KJ·m^−2^, the impact property of the foamed PP composites is higher than that of the unfoamed sample, but when the impact property is above 10.44 KJ·m^−2^, the impact property of the unfoamed sample is higher, which may provide important evidence for the toughening of foamed PP materials. In many research studies, it has also been reported that some foamed materials have shown an increase in impact property, while some foamed materials have shown a decrease in impact property, which provides a better explanation for this inconsistency [29,30,31]. In other words, the increase/decrease of impact property for the foamed polymer material may depend on the intrinsic toughness of the material. When the intrinsic toughness reaches a certain value, the impact property of the foamed material is decreased, but for lower values, the impact property of the material after foaming is increased.

Therefore, the key factor of the impact property for the foamed materials lies in the joint determination of the intrinsic properties of material and the toughening of cells. An empirical formula can be established to explain:

(5) $$\alpha_{k}=\alpha_{0}-\alpha_{1}+\alpha_{2}$$

where $\alpha_{0}$ is the impact property of the matrix, $\alpha_{1}$ represents the drop in impact property due to the introduction of cells, while $\alpha_{2}$ represents the increase in impact property due to the contribution of cells.

For the PP foamed composites, when the impact property is below 10.44 KJ·m^−2^, the introduction of cells compensates for the loss of toughness due to the reduction of the effective cross-sectional area. $\alpha_{2}$ > $\alpha_{1}$, so the impact property of material is improved. However, when the impact property is above 10.44 KJ·m^−2^, the toughening effect of cells is not equal to toughness reduction that arose from the effective cross-sectional area ($\alpha_{2}<\alpha_{1}$), which caused a decrease in the impact property of the material.

### 3.5. Effect of Cells on the Low-Temperature Toughness of PP Composites

In order to reflect the contribution of cells to the impact properties in foamed materials, the impact properties at −80 to 20 °C were analyzed. Here, samples of the foamed and unfoamed PP/POE (20 wt %) composites were chosen as the example.

**Figure 8:**
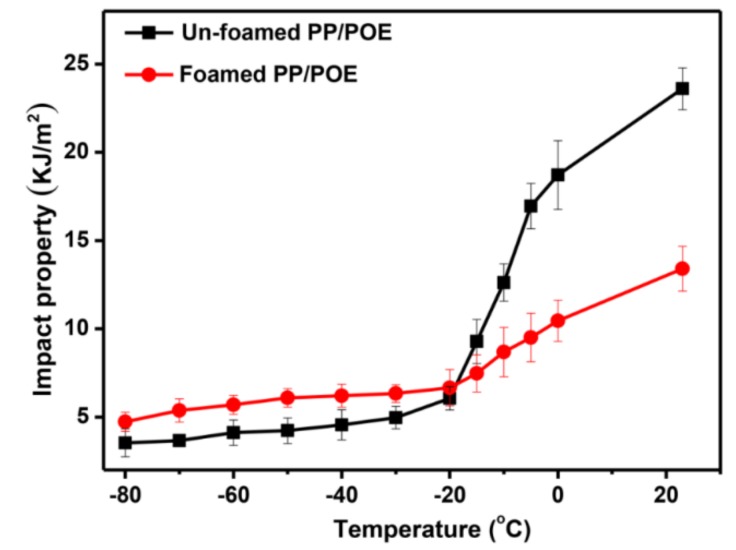
Analysis of low-temperature impact properties of PP/POE (20 wt %) composites.

**Figure 9:**
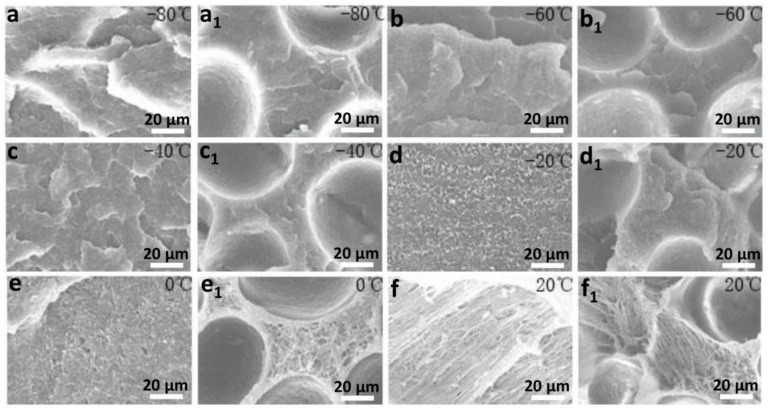
Microstructure of the foamed and unfoamed PP/POE composites at different temperatures: (**a**–**f**) unfoamed sample, (**a_1_**–**f_1_**) foamed sample.

Figure 8 shows that the brittle–ductile transition temperature of PP/POE composites is −20 °C. When the temperature was −80 to −20 °C, the impact property of the foamed or unfoamed samples all increased slowly, and the foamed sample had a higher value of impact property than that of the unfoamed sample. The increase in the impact property of the foamed sample is entirely due to the cell contribution below −20 °C; meanwhile, the fracture morphologies indicate that the PP molecular chain is brittle at low temperatures (shown in Figure 9a–d,a_1_–d_1_) with less contribution to impact property. When the temperature is higher than −20 °C, the impact property of the unfoamed sample rises faster than that of the foamed sample. At this time, the PP matrix material contributes a lot to the impact property, while the cells do little, which is further evidenced by the microstructures shown in Figure 9d–f,d_1_–f_1_.

According to Figure 9, in the temperature ranging from −80 to 20 °C, the impact fracture morphology is relatively smooth and exhibits typical brittle fracture characteristics whether it is a foamed or unfoamed sample. At this time, the polymer chains in the material are frozen and brittle at low temperature, which have little effect on the impact property when subjected to impact load [25]. The existence of cells is a key factor to increase the impact property of the foamed material. At −20 °C, the characteristics of the fracture for the foamed and unfoamed samples all became rough, and some microfibrils formed under the load, which indicates that this temperature (−20 °C) is the ductile–brittle transition temperature point of the samples. When the temperature was between −20 and 20 °C, the formation of microfibril gradually increased with the increase of temperature (shown in Figure 9d–f,d_1_–f_1_). At this time, the impact property was mainly attributed to the matrix material, so the impact property of the unfoamed sample was larger than that of the foamed sample. These results further explain the variation in Figure 6 and the feasibility of the empirical in Formula (5).

**Figure 10:**
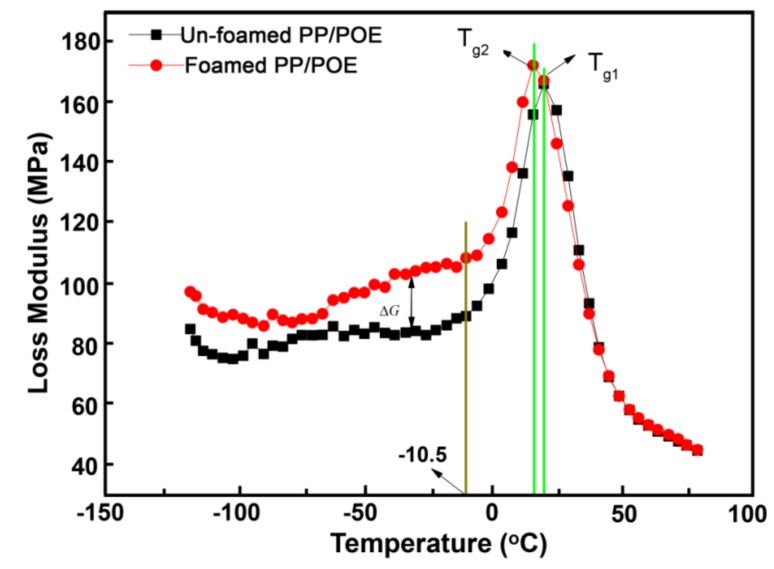
Loss modulus of the foamed and unfoamed PP (POE, 20 wt %) composites at different temperatures.

To further explain the impact properties of the foamed/unfoamed PP composites at low temperature (−80 to −20 °C), dynamic mechanical analysis (DMA) was conducted. Figure 10 shows that the loss modulus of the foamed PP composites was significantly higher than that of the unfoamed sample between −120 and −10.5 °C. Generally, the greater the loss modulus of a material, the better its viscosity and toughness [25,29]. Therefore, the toughness of the foamed PP composites was higher than that of the unfoamed sample between approximately −120 and −10.5 °C. In low temperature, the molecular chain segments in PP material can only vibrate slightly around a fixed position, and cannot be rearranged to release stress, resulting in the brittle state of the material [21]. In this status, the PP matrix material for the contribution of modulus and toughness is relatively small, and the increase of loss modulus and toughness of the foamed PP composites mainly arises from the contribution of cells. Moreover, it could be seen that the glass transition temperature (*T*_g_) of the PP composites shifts toward the low-temperature range after the introduction of cells; usually, a decrease in the transition temperature indicates an increase in toughness in terms of macromechanics. In a word, the introduction of cells has a certain role in increasing the toughness of the PP matrix. 

## 4. Conclusions

In conclusion, the foaming quality of the PP composites was obviously improved after the addition of TPR or POE, and there was no bubble phenomenon. Compared with the pure PP materials, the number of cells in the foamed PP composites increased significantly, reaching 7.54 × 10^6^ (TPR) and 12.5 × 10^6^ cell·cm^−3^ (POE), respectively, and the average diameter of cells was also significantly reduced, with values of 31.21 (TPR) and 25.42 μm (POE), respectively. Moreover, the influences of TPR or POE contents on the impact properties of PP composites were studied, and the results showed that the key factor of impact property for the foamed materials relied on the union of the intrinsic properties of the material and the toughening of cells. Particularly, the low-temperature toughness of PP composites showed that when the temperature was between −80 and −20 °C, the impact property of the foamed sample was higher than that of the unfoamed sample, and the increase in impact property was entirely due to the introduction of cells. When the temperature was in the range of −20 to 20 °C, the impact property of the unfoamed sample was much larger, which was due to the PP matrix contributing more to the impact property, while the cell did little at this temperature.

## Figures and Tables

**Table 1:** The density and diameter of the cells in foamed PP composites.

Sample	Cell Density (*N*_0_) (× 10^6^ cells·cm^−3^)	Cell Diameter ($\bar{D}$) (μm)	Cell Dispersion (*S_d_*) (μm)
PP	2.62	55.36	20.5
PP/TPR	7.54	31.21	12.3
PP/POE	12.5	25.42	8.1

**Table 2:** Crystallization data of the PP composites.

Sample	Initial Crystallization Temperature (°C)	Crystalline Peak Temperature (°C)	Crystallinity (*X_c_*) %
PP	106.6	113.3	43.1
PP/TPR	107.2	113.8	36.1
PP/POE	109.3	115.7	32.3
